# PATTERNS AND PREDICTORS OF ADULT GRANDCHILDREN’S PERCEPTIONS OF GRANDMOTHERS’ FAVORITISM AND DISFAVORITISM

**DOI:** 10.1093/geroni/igae098.2732

**Published:** 2024-12-31

**Authors:** Destiny Ogle, J Jill Suitor, Megan Gilligan, Reilly Kincaid

**Affiliations:** Purdue University, West Lafayette, Indiana, United States; Purdue University, West Lafayette, Indiana, United States; University of Missouri, Columbia, Missouri, United States; Purdue University, West Lafayette, Indiana, United States

## Abstract

Scholarship on later-life families has shown that parents differentiate among their adult children across a range of relational dimensions and that perceptions of parental favoritism and disfavoritism have consequences for children’s well-being. Far less is known about patterns of favoritism and disfavoritism in other intergenerational ties. We extend this line of research by exploring adult grandchildren’s perceptions of their grandmothers’ favoritism and disfavoritism using mixed-methods data collected from 229 adult grandchildren nested within 94 families collected as part of the Within-Family Differences Study T3. On average, grandchildren were 28 years old and grandmothers were 92 years old. Eighty percent perceived that their grandmothers were most emotionally close to particular grandchildren, and 64% perceived that their grandmothers had the most conflict with particular grandchildren. Twenty-five percent perceived they were the closest to their grandmothers, and 12% perceived that they had the most conflict. MLM analyses revealed that respondents who were first-borns and in greater contact with grandmothers were most likely to report being the closest. Those most likely to report having the greatest conflict with their grandmothers were unmarried, did not share grandmothers’ values, and provided grandmothers with care during illnesses. Qualitative analyses shed light on how this set of factors played salient roles in grandmothers’ favoritism and disfavoritism. These findings indicate that adult grandchildren are as likely as adult children to perceive favoritism and disfavoritism, and that the factors predicting perceiving oneself as favored or disfavored by grandmothers greatly mirror those found in studies of adult children and older parents.

